# Cross-species amplification of 36 cyprinid microsatellite loci in *Phoxinus phoxinus *(L.) and *Scardinius erythrophthalmus *(L.)

**DOI:** 10.1186/1756-0500-2-248

**Published:** 2009-12-14

**Authors:** Johannes Holmen, Leif A Vøllestad, Kjetill S Jakobsen, Craig R Primmer

**Affiliations:** 1 Centre for Ecological and Evolutionary Synthesis (CEES), Department of Biology, University of Oslo, PO Box 1050 Blindern, N-0316 Oslo, Norway; 2 Division of Genetics and Physiology, Department of Biology, 20014 University of Turku, Finland

## Abstract

**Background:**

To conduct phylogeographic or population genetic studies, an adequate number of DNA markers for the focal species are required. Due to severe unavailability of genotype markers of any kind for the species Eurasian minnow (*Phoxinus phoxinus *L.) and rudd (*Scardinius erythrophthalmus *L.), we set out to attempt cross-amplification of a set of microsatellite loci from related species.

**Findings:**

We tested 36 cyprinid microsatellite loci for cross-species amplification in minnow and rudd. Fifteen species-locus combinations produced amplifications in minnow, seven being polymorphic, while 18 combinations amplified in rudd, nine of these being polymorphic.

**Conclusions:**

The positive cross-species amplifications present potential contributions to the establishment of genetic marker sets for population genetics studies of the two focal species.

## Findings

Microsatellites are widely used for population genetics purposes, especially when the scope of the study involves comparing closely related individuals. This is mainly due to their high mutation rates and to the potential of acquiring large amounts of data through relatively labour-thrifty multi-marker panel runs on capillary electrophoresis sequencers. However, utilization of microsatellites demands knowledge about their flanking sequences generated through library construction and/or PCR cloning approaches [1] to construct adequately sized annealing primer pairs. The flanking regions of microsatellites usually mutate at a much slower rate than the microsatellites themselves and will in many cases be identical across a species' range of distribution. They may even be conserved well enough through evolution to serve as primer templates for closely related species (see e.g. [2-4]).

The diverse family Cyprinidae, the most species-rich family of all vertebrates, has been paid only limited attention in population genetics studies. In the few studies available, the primary focus has been on a few species that have shared the status of being either commercially important or popular game fish; exemplified by studies on common carp (*Cyprinus carpio *L.) [5-7], goldfish (*Carassius auratus *L.) [8], European chub (*Leuciscus cephalus *L.) [9] and the genetic model species zebrafish (*Danio rerio *Hamilton) [10]. Therefore, for the great majority of cyprinids genetic markers are unavailable. In an earlier study, Holmen *et al*. [4] established a platform for optimization of microsatellite markers for six different cyprinids based on cross-species amplification of markers that initially were developed for *D. rerio *and central stoneroller (*Campostoma anomalum *Rafinesque). In this subsequent study, the platform was extended for two of the six species; Eurasian minnow (*Phoxinus phoxinus *L.) and rudd (*Scardinius erythrophthalmus *L.). We tested 36 microsatellite loci developed for the five cyprinid species fathead minnow (*Pimephales promelas *Rafinesque) [11], silver barb (*Barbonymus gonionotus *Bleeker) [12,13], common carp (*Cyprinus carpio carpio *L.) [14], *Anaecypris hispanica *(Steindachner) [15], and goldfish (*Carassius auratus auratus *L.) [16] for amplification of minnow and rudd DNA. Primer design incorporated the original primers with the addition of GTTT to the 5' end of one primer of each pair facilitating reasonably consistent adenylation of the 3' end of the forward primer [17] (see Additional file 1). We tested two samples of each focal species, yielding a total of 144 PCR reactions. To ascertain the polymorphism of a potential microsatellite locus, it is advisable to include more than only two samples of the focal species; however, we selected samples from populations situated far apart to increase the probability of polymorphism detection; minnow samples were from Norway and Spain, rudd from Norway and Italy.

We extracted genomic DNA from ethanol preserved fin tissue using a salt extraction protocol outlined by Aljanabi & Martinez [18]. Further, we performed PCR reactions on MJ Research PTC-100/PTC-200, Techne or Biometra thermal cyclers. In the total volume of 10 μl, PCR reactions contained 20-100 ng genomic DNA, 20 μM dNTP, plus 1 μM fluorescently labelled dUTPs (R110, R6G or TAMRA FdUTP; Applied Biosystems), 0.5 μM of each primer, 1× BioTaq buffer (160 mM (NH_4_)_2_SO_4_, 670 mM Tris-HCl, 0.1% Tween-20; all buffer concentrations), 1.5 mM MgCl_2_, and 0.1 units of BioTaq DNA polymerase (Bioline). PCR protocols were constructed with annealing temperatures and durations of incubations from published recommendations for the source species in mind. However, all PCR reactions were transformed to 'touchdown' procedures; starting with a relatively high annealing temperature, gradually decreasing it for each cycle and eventually keeping a fixed annealing temperature for a number of cycles towards the end. Details of the PCR protocols for all markers are given in Additional file 1.

PCR products were pooled with a loading buffer and size standard mix (MegaBACE 10× Running buffer and MegaBACE ET400-R Size Standard, GE Healthcare, formerly Amersham Biosciences) and electrophoresed using a MegaBACE 1000 sequencer (GE Healthcare). Genotypes were scored using Genetic Profiler 1.5 (GE Healthcare). Scoring results were classified according to their amplification quality level, as outlined in Primmer & Merilä [19]: 1: 1 or 2 alleles observed in a single individual, with little stuttering observed; 2: 1 or 2 alleles, moderate stutter; 3: 1 or 2 alleles, considerable stutter; 4: multiple bands and/or smear; 5: no amplification. Due to the possible confusion between true microsatellite alleles and other amplifications, bands having no trace of a weaker band one repeat below were included in category 4, even when only one or two bands were observed. Note that no positive controls were used in these runs.

Thirty-three out of the total of 72 heterologous locus-species combinations resulted in products of amplification quality 3 or better (Table 1). Of these successful combinations, 15 (seven polymorphic) were recorded for minnow and 18 (nine polymorphic) for rudd. Interestingly, all eight successful amplifications with *C. carpio *loci were polymorphic for both target species, while only 24% of the remaining amplifying loci were polymorphic. Average amplification successes in Holmen *et al*. [4] were 40% in rudd and 49% in minnow, while the corresponding figures in the current study were 50% and 42%, respectively. Some of the amplified loci were later optimized for population genetics studies in minnow (Holmen *et al*., in prep.), the selection being based on the number of alleles revealed in this study, amplification quality, and, in order to fit into an already half completed panel, the size range in which alleles appeared. Thus, only *MFW1*, *MFW17*, and *GF11 *have been further optimized and amplified in 1660 minnows from 72 sampling sites across Europe (Table 2). These three loci produced reasonably strong, unambiguous peaks after some optimization, and were included in the population studies. However, *GF11 *proved to exhibit very little variation; in fact it was monomorphic in 55 sampling sites, and thus the amount of genetic information from this locus was very limited. Within-population deviations from Hardy-Weinberg equilibrium were tested for using Genepop [20,21]. For these tests, only those 44 sampling sites that consisted of at least 17 individuals were included. *MFW1 *was polymorphic for all of these sampling sites, while *MFW17 *was polymorphic in all but one. For these two loci, none and one, respectively, of the samples deviated from Hardy-Weinberg equilibrium at the Bonferroni-adjusted 0.05 significance level. For *GF11*, only eleven samples were polymorphic, and out of those one was in Hardy-Weinberg disequilibrium. To specifically test for the presence of null alleles, ML-NullFreq [22] was employed. Using the Bonferroni-adjusted 0.05 significance level, one and four out of the 44 samples indicated the presence of null alleles in *MFW1 *and *MFW17*, respectively. For *GF11*, six out of the eleven polymorphic sampling sites indicated the presence of null alleles, further emphasizing the limited value of this locus in population genetics studies. Unfortunately, further information of the tested loci is presently unavailable for *S. erythrophthalmus*.

**Table 1:** Details of cross-species amplification of 36 cyprinid microsatellites in *P. phoxinus *and *S. erythrophthalmus*

			**Focal species**					
			
**Source species**	**Locus**	**Repeat motif**	***P. phoxinus***			***S. erythrophthalmus***		
				
			**A**	**Size (bp)**	**Q**	**A**	**Size (bp)**	**Q**
				
	Ppr101	AC	0	-	5	1	395	1
	Ppr102	AC	0	-	5	0	-	5
	Ppr103	AC	0	-	5	0	-	5
*P. promelas*	Ppr104	AC	1	122	2	0	-	5
	Ppr105	AC	0	-	4	1	222	1
	Ppr106	AC	0	-	4	0	-	5
	Ppr107	ACAG	1	200	2	1	266	1
				
	Bgon8	AC	0	-	5	2	138-178	2
	Bgon13	GT	0	-	4	0	-	5
	Bgon17	AC	1	149	3	0	-	5
*B. gonionotus*	Bgon22	TCC	0	-	5	4	103-151	3
	Bgon69	TG	0	-	5	1	260	2
	Bgon75	AC	1	78	3	0	-	5
	Bgon79	CA	0	-	4	3	154-208	2
				
	MFW1	CA	4	166-226	2	3	172-178	2
	MFW5	CA	0	-	5	2	103-107	2
	MFW17	CA	2	191-195	1	2	183-187	2
*C. carpio*	MFW18	CA	0	-	5	0	-	5
	MFW19	CA	2	222-226	2	4	191-203	2
	MFW24	CA	2	137-161	2	0	-	5
	MFW28	CA	0	-	5	0	-	5
				
	II04	GT	0	-	5	0	-	5
	IV04	CA*	0	-	5	1	168	3
	IV34	CA*	1	108	1	1	109	1
	IV46	TG*	0	-	5	0	-	5
*A. hispanica*	X44	CA*	1	148	2	1	148	2
	XII02	CA*	1	88	2	0	-	5
	XIII40	GT*	0	-	4	0	-	4
	XIV13	GT/GA*	0	-	5	0	-	4
	XIV31	CA*	2	93-149	2	2	93-147	2
	XV28	CA*	0	-	5	2	160-172	3
				
	GF1	TG	2	90-226	1	0	-	4
	GF11	TG*	3	149-161	2	1	161	3
*C. auratus*	GF17	TG	1	114	1	0	-	5
	GF20	TG	0	-	5	0	-	5
	GF29	TG*	0	-	5	1	109	1

Number of alleles recorded (A), size (bp), and quality of the amplified product (Q: 1, little stutter; 2, moderate stutter; 3, considerable stutter; 4, multiple bands or smear; 5, no amplification) are given. Asterisks denote non-continuous repeat motif sequences.

**Table 2:** Population genetics parameters for the loci *MFW1*, *MFW17 *and *GF11 *in *P. phoxinus*, based on amplifications in 1660 individuals from 72 sampling sites across Europe

**Locus**	**Number of alleles**	**N_E_**	**H_E_**	**H_O_**	**F_IS_**
					
MFW1	31	6.52	0.85	0.66	-0.02
					
MFW17	38	4.15	0.76	0.53	0.02
					
GF11	3	1.08	0.07	0.05	0.26

The likelihood that primer pairs developed for one species should amplify in a second species is higher the more closely related the two species are. On that general basis, one can assume the relative success rate among a number of cross-species amplification attempts. Cyprinidae taxonomy is rather complex. Although the family has traditionally been organized into several subfamilies, each comprising one or more lineages which in turn include a number of genera, and most lineages and genera are generally accepted as being monophyletic, there is controversy regarding the monophyly of some subfamilies [23]. Thus, we had few obvious expectations regarding amplification successes in the present cross-species study. *S. erythrophthalmus*, *P. phoxinus, P. promelas*, and *A. hispanica *all belong to the subfamily Leuciscinae, but Hänfling & Brandl [24] considered the genus *Phoxinus *to be a sister taxon to a Leuciscinae-Alburninae lineage. *C. carpio*, *C. auratus*, and *B. gonionotus *all belong to the Cyprininae subfamily. *P. promelas *and *A. hispanica *loci were thus expected to amplify with a reasonably high rate in *S. erythrophthalmus *and slightly lower in *P. phoxinus*, and indeed, the success rates, defined as the proportions that produced peaks with amplification quality 3 or better, were 43% versus 29% and 50% versus 40% in favour of *S. erythrophthalmus *for the two source species, respectively. Loci from the Cyprininae subfamily were expected to produce a lower success rate in both target species. This was not the case, however, as amplification success ranged from 29% to 57%. Notably, the number of loci examined is too low for any differences observed to be statistically significant. The results should therefore not be regarded as a contribution to the lineage discussion within Cyprinidae.

The present and our previous study [4] points out the usefulness of cross-species amplification of microsatellites in Cyprinidae to establish markers for population genetics studies. More specifically, the findings in these two papers have provided the authors with a useful set of markers for phylogeography and population genetics studies of the minnow and will hopefully contribute to fellow researchers' related work as well.

## Competing interests

The authors declare that they have no competing interests.

## Authors' contributions

JH carried out the molecular genetics laboratory work and drafted the manuscript. LAV conceived the study. KSJ helped design laboratory procedures. CRP had the major input to the design of the study. All authors read, contributed to and approved the final manuscript.

## Supplementary Material

Additional file 1 Primer sequences and PCR protocol details of all loci investigated Click here for file
