# Supplementary material for: Cross-species amplification of 36 cyprinid microsatellite loci in Phoxinus phoxinus (L.) and Scardinius erythrophthalmus (L.)
Source: BMC Res Notes. 2009 Dec 14;2:248. doi: 10.1186/1756-0500-2-248 (PMC2801513; doi:10.1186/1756-0500-2-248)
Supplement: Additional file 1 — Primer sequences and PCR protocol details of all loci investigated [file 1756-0500-2-248-S1.DOC]

**Additional file 1.** **Primer sequences and PCR protocol details of all loci investigated**

| **Species** |  | **Locus** |  | **Primer sequences, forward and reverse** |  | **PCR protocols** | | | | | | |
| --- | --- | --- | --- | --- | --- | --- | --- | --- | --- | --- | --- | --- |
|  |  |  |  |  |  | **T_A_/cycle details** |  | **Cycle step durations** | | | | |
|  |  |  |  |  |  |  |  | **Denaturation** |  | **Annealing** |  | **Extension** |
| *Pimephales promelas*^1^ |  | Ppr101 |  | 5’-TCCTTGAAATCAGAGGACTG-3’  5’-GTTTCGAAACATCAGTACGCAAGC-3’ |  | 52/46/32 |  | 30s |  | 30s |  | 30s |
|  |  | Ppr102 |  | 5’-GGGAACCATGCAGGACAGGC-3’  5’-GTTTAATTCCTGCACCTTGTCTGCC-3’ |  |  |  |  |  |  |  |  |
|  |  | Ppr103 |  | 5’-AGCATTGGTTTCCTGAACCC-3’  5’-GTTTGCACTGAATAACGTGTGAAAGC-3’ |  |  |  |  |  |  |  |  |
|  |  | Ppr104 |  | 5’-TGGTTCTTCTCAGATGCTGTTG-3’  5’-GTTTCGGCTCTGGGAGGAGACG-3’ |  |  |  |  |  |  |  |  |
|  |  | Ppr105 |  | 5’-CTGTGCCCTGTCTACTAGCG-3’  5’-GTTTATACTCTGTAGTGTCACCAGG-3’ |  |  |  |  |  |  |  |  |
|  |  | Ppr106 |  | 5’-CCCACATACTTGCTCGCTCG-3’  5’-GTTTGGCTTTAAATGACCTTCAACAAG-3’ |  |  |  |  |  |  |  |  |
|  |  | Ppr107 |  | 5’-TATGTGGTGGAATGGGAGTC-3’  5’-GTTTGGCTGAGAGAATAAGCTTGC-3’ |  |  |  |  |  |  |  |  |
| *Barbonymus gonionotus*^2^ |  | Bgon8 |  | 5’-GATAGATTCATTCAGCTGGCTG-3’  5’-GTTTACGTTTGTGTATGTGGATGTGC-3’ |  | 60/50/30 |  | 30s |  | 30s |  | 30s |
|  |  | Bgon13 |  | 5’-CCCGTGCAATTCAATATG-3’  5’-GTTTAAGTAGCACAGATGTGAGG-3’ |  | 53/47/30 |  | 30s |  | 30s |  | 30s |
|  |  | Bgon75 |  | 5’-CTGGTAAAGACTTCAGATGC-3’  5’-GTTTGCATGCAAAATGAGAAAGGCT-3’ |  |  |  |  |  |  |  |  |
|  |  | Bgon79 |  | 5’-GCCAGACTGGAGCGAGG-3’  5’-GTTTGTTCGGTGAAGCCATGAGG-3’ |  |  |  |  |  |  |  |  |
|  |  | Bgon17 |  | 5’-TTACAAGGGGTTACATACTG-3’  5’-GTTTCAGTCTCATATTTGAAAGCAG-3’ |  | 49/43/30 |  | 30s |  | 30s |  | 30s |
|  |  | Bgon22 |  | 5’-TCTTGTTGATCACACGGACG-3’  5’-GTTTGTGACTGTATCAATGAGTCTG-3’ |  |  |  |  |  |  |  |  |
|  |  | Bgon69 |  | 5’-GCAAAGGTTCTGTCAAGG-3’  5’-GTTTGTATCCAGAAACATGTTCAG-3’ |  |  |  |  |  |  |  |  |
| *Cyprinus carpio*^3^ |  | MFW1 |  | 5’-GTCCAGACTGTCATCAGGAG-3’  5’-GTTTGAGGTGTACACTGAGTCACGC-3’ |  | 53/45/37 |  | 40s |  | 45s |  | 50s |
|  |  | MFW5 |  | 5’-GAGATGCCTGGGGAAGTCACGC-3’  5’-GTTTAAAGAGAGCGGGGTAAAGGAG-3’ |  |  |  |  |  |  |  |  |
|  |  | MFW17 |  | 5’-CTCAACTACAGAGAAATTTCATC-3’  5’-GTTTGAAATGGTACATGACCTCAAG-3’ |  |  |  |  |  |  |  |  |
|  |  | MFW18 |  | 5’-GTCCCTGGTAGTGAGTGAGT-3’  5’-GTTTGCGTTGACTTGTTTATACTAG-3’ |  |  |  |  |  |  |  |  |
|  |  | MFW28 |  | 5’-GATCCCTTTTGAATTTTCTAG-3’  5’-GTTTACAGTGAGGTCCAGAAGTCG-3’ |  |  |  |  |  |  |  |  |
|  |  | MFW19 |  | 5’-GAATCCTATCATGCAAAC-3’  5’-GTTTGCACAAACTCCACATTGTGCC-3’ |  | 56/48/35 |  | 40s |  | 45s |  | 50s |
|  |  | MFW24 |  | 5’-GCTCCAGATTGCACATTATAG-3’  5’-GTTTCTACACACACGCAGAGCCTTC-3’ |  | 54/46/35 |  | 40s |  | 45s |  | 50s |
| *Anaecypris hispanica*^4^ |  | II04 |  | 5’-GAGTTGATTGCACTTGGTTAGG-3’  5’-GTTTTGACTTAAACCCCAATCC-3’ |  | 60/50/40 |  | 30s |  | 30s |  | 30s |
|  |  | IV04 |  | 5’-TGCAGAGATGGATGAACTGAGC-3’  5’-GTTTATACGGCACGACATGTTTCC-3’ |  |  |  |  |  |  |  |  |
|  |  | IV34 |  | 5’-CCAAGGTTACTTTGCATTCG-3’  5’-GTTTCAATGATAACCGTGCCTTCC-3’ |  |  |  |  |  |  |  |  |
|  |  | IV46 |  | 5’-TTTTCCCACTTTACTCTCCCAC-3’  5’-GTTTCTGCGACCTGTCATGATATG-3’ |  |  |  |  |  |  |  |  |
|  |  | X44 |  | 5’-GTTTGCACCTTCATTCAGGC-3’  5’-ACAGTCGTATGGAAACTGGTACG-3’ |  |  |  |  |  |  |  |  |
|  |  | XII02 |  | 5’-AGTTTTAACACTGGTGGACTGG-3’  5’-GTTTGCTGTTGTTTGTTAATCAGAGTGC-3’ |  |  |  |  |  |  |  |  |
|  |  | XIII40 |  | 5’-CTTAGTCAGCCACAGTCGTATG-3’  5’-GTTTCTTCATTCAGGCTTTAGGCA-3’ |  |  |  |  |  |  |  |  |
|  |  | XIV13 |  | 5’-GAGCAAAGAGTTTGAAAGAGAGG-3’  5’-GTTTCTTTTCCACTGGCACTGTATG-3’ |  |  |  |  |  |  |  |  |
|  |  | XIV31 |  | 5’-GCAGCCTGAACCATAAACG-3’  5’-GTTTCGGAATATTTCCTCCCTACC-3’ |  |  |  |  |  |  |  |  |
|  |  | XV28 |  | 5’-AGTTTTAACACTGGTGGACTGG-3’  5’-GTTTGAACAAGTGAAAATAAGACAAGTATGC-3’ |  |  |  |  |  |  |  |  |
| *Carassius auratus*^5^ |  | GF1 |  | 5’-ATGAAGGGTAGGAAAAGTGTGA-3’  5’-GTTTCAGGTTAGGGAGAAGAAGGAAT-3’ |  | 58/50/37 |  | 30s |  | 20s |  | 30s |
|  |  | GF11 |  | 5’-GCATTCTTCTGGTTGGTTCT-3’  5’-GTTTACCACCACATTCAGCAGTCC-3’ |  |  |  |  |  |  |  |  |
|  |  | GF17 |  | 5’-GGAACTAGAGCCCACTGACA-3’  5’-GTTTGCATTTGGGAGACGATA-3’ |  |  |  |  |  |  |  |  |
|  |  | GF20 |  | 5’-AAAGTAAAAGTAATTCTCAAAAT-3’  5’-GTTTATTGGAGTAAAGGCTGATG-3’ |  |  |  |  |  |  |  |  |
|  |  | GF29 |  | 5’-ATGCTAGGTGACTGTTTGT-3’  5’-GTTTCACCTCCACTCCTAATAAT-3’ |  |  |  |  |  |  |  |  |

All PCR protocols were initiated with a 3 min denaturation at 94°C followed by a ’touchdown’ procedure, employing denaturation at 94°C, annealing temperature (T_A_) decreasing by 0.5°C per cycle for a number of cycles until reaching a plateau where T_A_ was constant through all remaining cycles, and extension at 72°C, and then terminated after a final 5 min (2 min for all *GF* loci) elongation at 72°C. The column ‘T_A_/cycle details’ denote first figure as initial T_A_, second figure as plateau T_A_, and third figure as total number of cycles. Further PCR optimizations for Holmen *et al.* (in prep.) can be obtained from the corresponding author. Primer designs are based on those described in ^1^Ardren *et al.* (2002), ^2^McConnell *et al.* (2001), ^2^Kamonrat *et al.* (2002), ^3^Crooijmans *et al.* (1997), ^4^Salgueiro *et al.* (2003), and ^5^Zheng *et al.* (1995). GenBank accession numbers can be obtained in the respective references.
